# The role of peripheral white blood cell counts in the association between central adiposity and glycemic status

**DOI:** 10.1038/s41387-024-00271-9

**Published:** 2024-05-17

**Authors:** Fengqiong Liu, Yanni Li, Wanxin Li, Ruimei Feng, Hongwei Zhao, Jun Chen, Shanshan Du, Weimin Ye

**Affiliations:** 1 Department of Epidemiology and Health Statistics, Fujian Provincial Key Laboratory of Environment Factors and Cancer, School of Public Health, Fujian Medical University, Fuzhou, China; 2 Department of Epidemiology and Biostatistics, School of Public Health, Texas A&M University, College Station, TX USA; 3 Department of Medical Epidemiology and Biostatistics, Karolinska Institutet, Stockholm, Sweden

**Keywords:** Epidemiology, Nutrition disorders, Diabetes

## Abstract

**Aims:**

Although central adiposity is a well-known risk factor for diabetes, the underlying mechanism remains unclear. The aim of this study was to explore the potential mediation role of circulating WBC counts in the association between central adiposity and the risk of diabetes.

**Materials and methods:**

A cross-sectional study was conducted using data from the Fuqing cohort study, which included 6,613 participants aged 35–75 years. Logistic regression analysis and Spearman’s rank correlation analysis were used to examine the relationships between waist-to-hip ratio, WBC counts and glycemic status. Both simple and parallel multiple mediation models were used to explore the potential mediation effects of WBCs on the association of waist-to-hip ratio with diabetes.

**Results:**

The study revealed a positive relationship between waist-to-hip ratio and risk of prediabetes (*OR* = 1.53; 95% *CI*, 1.35 to 1.74) and diabetes (*OR* = 2.89; 95% *CI*, 2.45 to 3.40). Moreover, elevated peripheral WBC counts were associated with both central adiposity and worsening glycemic status (*P* < 0.05). The mediation analysis with single mediators demonstrated that there is a significant indirect effect of central adiposity on prediabetes risk through total WBC count, neutrophil count, lymphocyte count, and monocyte count; the proportions mediated were 9.92%, 6.98%, 6.07%, and 3.84%, respectively. Additionally, total WBC count, neutrophil count, lymphocyte count, monocyte count and basophil count mediated 11.79%, 11.51%, 6.29%, 4.78%, and 1.76%, respectively, of the association between central adiposity and diabetes. In the parallel multiple mediation model using all five types of WBC as mediators simultaneously, a significant indirect effect (*OR* = 1.09; 95% *CI*, 1.06 to 1.14) were observed, with a mediated proportion of 12.77%.

**Conclusions:**

Central adiposity was independently associated with an elevated risk of diabetes in a Chinese adult population; levels of circulating WBC may contribute to its underlying mechanisms.

## Introduction

Diabetes mellitus (DM), a major endocrine disorder and chronic metabolic disease, is a serious threat to human health [1]. According to the latest data from the International Diabetes Federation (IDF), approximately 140.9 million diabetic subjects were diagnosed in China in 2021, and this number is predicted to increase to 174.4 million by 2040 [2]. A large body of evidence has shown that diabetes may lead to various potentially life threatening micro-vascular and macro-vascular complications [3–5].

Obesity, both general and abdominal obesity, is most likely the main risk factor for the development of type 2 diabetes (T2DM) [6, 7]. Moreover, accumulating studies demonstrate that abdominal obesity, as assessed by waist-to-hip ratio (WHR), is more pathogenic and consequently is more closely associated with T2DM than general obesity as quantified by BMI [8, 9]. However, the underlying mechanism between obesity and diabetes is complicated and remains to be illustrated. Inflammation might be one potential biological pathway.

It has been proposed that increased adiposity, particularly central adiposity, contributes directly toward an increase in systemic inflammation with the production of various inflammatory cytokines by adipocytes [10, 11]. Peripheral white blood cells (WBC), including neutrophils (NEUT), lymphocytes (LYM), monocytes (MNC), eosinophils (EOS), and basophils (BAS), are subclinical low-grade inflammatory indicators. Accumulating evidence has validated that subclinical low-grade inflammation plays a contributory role in the pathogenesis of T2DM [12]. Although several studies have investigated the effect of anthropometric measurements on inflammatory markers, studies using circulating white cell types as inflammation indicators are not common. Meanwhile, accumulating evidence reveals that elevated total white cell count is associated with higher risk of glucose metabolism disorders, such as diabetes [13, 14].

We conducted a cross-sectional study to investigate the relationship between central adiposity and glycemic status. Additionally, we explored whether and to what extent the association was mediated by total and distinct different WBC counts, using both simple and multiple mediation analysis.

## Methods

### Study design and participants

A cross-sectional study was conducted using baseline data collected from the Fuqing cohort study from July 2020 to June 2021 in Gaoshan town. The Fuqing cohort study is a prospective multi-purpose research program conducted by the Fujian Medical University and the local government of Fuqing City, located in Fujian province, Southeast China. The Fuqing cohort study investigates the natural history and risk factors of chronic non-communicable diseases, including cancer, diabetes, and fatty liver, among the Chinese population residing in the Southeast coastal region of China. Detailed information on sociodemographics (age, sex, level of education, and employment status), lifestyle factors (smoking, alcohol drinking, and tea drinking), history of chronic diseases, and history of taking medicine was collected through face-to-face questionnaires by trained interviewers. In addition, most participants underwent physical examination and provided biological samples. The inclusion criteria for this study were as follows: (1) aged 35-75 years; (2) residents of Fuqing city. The exclusion criteria were as follows: (1) participants with a history of cancer or type 1 diabetes mellitus; (2) participants with hypoglycemia at baseline. A total of 6790 participants were initially included. In addition, we excluded 177 participants with missing values for waist circumference, blood parameters, or glycemic status indicators. Finally, a total of 6,613 subjects were included in the final analysis. This study was approved by the ethical committee of Fujian Medical University (Approval number, 2017–07 and 2020–58), and the study protocol conforms to the ethical guidelines of the 1975 Declaration of Helsinki and all participants provided written informed consent.

### Definition of central adiposity

Anthropometric measurements including waist and hip circumference were obtained using standardized methods by trained examiners. Waist circumference was measured in centimeters with a nonstretchable tape held at the midpoint between the lower margin of the least palpable rib and the top of the iliac crest, and hip circumference was measured around the widest portion of the buttocks. WHR was calculated as waist circumference/hip circumference. According to the World Health Organization (WHO) cut-off points for substantially increased risk of metabolic complications, central adiposity was defined as WHR ≥ 0.90 in men and ≥ 0.85 in women [15].

### Measurement of peripheral WBC counts

Venous blood sample of each participant was obtained. WBC including neutrophil, lymphocyte, monocyte, eosinophil, and basophil were measured using standard laboratory procedures (fully automated blood cell analyzer, Xs-1000i, Sysmex, Osaka, Japan). Total WBC was defined as the sum of all five types of white blood cells.

### Definition of normal glucose, prediabetes, and diabetes

According to the WHO criteria [16], participants were classified into three groups: normoglycemia, prediabetes, and diabetes. Prediabetes was defined as fasting blood glucose 110 mg/dL (6.1 mmol/L) to 125 mg/dL (6.9 mmol/ L), and oral glucose tolerance test: two hour blood glucose <140 mg/dL (7.8 mmol/L), or fasting blood glucose <126 mg/dL (7.0 mmol/L), and oral glucose tolerance test: two-hour blood glucose 140 mg/dL (7.8 mmol/L) to 199 mg/dL (11.0 mmol/L) in participants without a history of diabetes. Diabetes was defined as a previous physician diagnosis of diabetes, or current use of diabetes medication, or having one or more of the following: (1) fasting blood glucose ≥126 mg/dL (7.0 mmol/L); (2) oral glucose tolerance test: two-hour blood glucose ≥200 mg/dL (11.1 mmol/L).

### Assessment of covariates

Demographic data, including age, sex, education, occupation, as well as, lifestyle behaviors (smoking, alcohol drinking, tea drinking), status of chronic disease (hypertension and hyperlipidemia), and history of taking lipid-lowering drugs were collected via standardized questionnaires at baseline. Hypertension was defined as one or more of the following: (1) self-reported or doctor-diagnosed hypertension, (2) current treatment with antihypertensive agents, (3) systolic blood pressure ≥140 mmHg and/or diastolic blood pressure ≥90 mmHg. Hyperlipidemia was defined as one or more of the following: total cholesterol (TC) ≥ 6.2 mmol/L, triglyceride (TG) ≥ 2.3 mmol/L, high-density lipoprotein cholesterol (HDL-c) <1.0 mmol/L, or low-density lipoprotein cholesterol (LDL-c) ≥4.1 mmol/L.

### Sensitivity analysis

Sensitivity analysis was performed by excluding participants who used antibiotic medication for infection in the past 6 months, and participants with abnormal white blood cell count (<2.5% or > 97.5% of the distribution) to estimate the association between WBC counts and glycemic status, and the mediation effect of WBC counts in the association between WHR and glycemic status.

### Statistical analysis

Demographic and clinical characteristics were first presented for the normal glycemic group, prediabetic, and diabetic groups. Categorical variables were expressed as numbers (percentages) and tested for association with glycemic status using Chi-squared tests. Continuous variables were presented as medians and interquartile ranges (IQR) and tested using Kruskal-Wallis rank-based tests, owing to their non-normal distributions.

In the analysis investigating the effect of WHR, or WBC counts on the glycemic status, we use age, sex, education, occupation, smoking, alcohol drinking, tea drinking, hypertension, hyperlipidemia, and lipid-lowering drugs as covariates. Separate logistic regression models were used to evaluate the associations of WHR or WBC counts with prediabetes and diabetes. Furthermore, Spearman’s rank correlation analysis was conducted to quantify the relationship between WHR and WBC counts. Statistical analyses were conducted by using Stata (version 16.0).

Mediation analysis was also conducted to explore whether the effect of WHR on diabetes was mediated by white blood cells, which include neutrophils, lymphocytes, monocytes, eosinophils, and basophils. Both simple and multiple mediation analyses were employed to assess the roles of different WBC counts in the association between WHR and diabetes. In the mediation analysis, we decomposed the total effects into natural direct and indirect effects [17, 18] using CMAverse [19], an R package that provides a suite of functions for causal mediation analysis. The 95% confidence intervals (*CI*s) for indirect effects were evaluated using bias-corrected CIs from 10000 bootstrapping samples (Rstudio version 4.2.2).

## Results

### Characteristics of the study population

The demographic characteristics of 6613 subjects are described in Table 1. Compared to participants with normal glucose level, participants in the prediabetes or diabetes group were more likely to be older, less educated, and have higher BMI, waist circumference, and waist-to-hip ratio. The distribution of sex, occupation, smoking, tea drinking, hypertension, hyperlipidemia, and lipid-lowering drugs were also different among the groups. Additionally, the total and different white blood cell counts, including neutrophils, lymphocytes, monocytes, and basophils, were higher in prediabetes and diabetes group than those in the control group (*P* < 0.01). The distribution of white blood cells is presented in Supplementary Fig. 1.

**Table 1:** Basic characteristics of study participants according to glycemic status.

Variables	Normoglycemia (*n* = 3789)	Prediabetes (*n* = 1625)	Diabetes (*n* = 1199)	*P*-value
Age (years)				**<0.01**
<60	2376 (62.71)	826 (50.83)	457 (38.12)	
≥60	1413 (37.29)	799 (49.17)	742 (61.88)	
Sex				**<0.01**
Male	1420 (37.48)	532 (32.74)	414 (34.53)	
Female	2369 (62.52)	1093 (67.26)	785 (65.47)	
BMI (kg/m^2^)				**<0.01**
<18.5	101 (2.67)	37 (2.28)	17 (1.42)	
18.5–24	2050 (54.10)	730 (44.92)	431 (35.95)	
24–28	1320 (34.84)	639 (39.32)	509 (42.45)	
≥28	318 (8.39)	219 (13.48)	242 (20.18)	
Waist circumference (cm)				
Male				**<0.01**
<90	1022 (26.97)	335 (20.62)	229 (19.10)	
≥90	398 (10.50)	197 (12.12)	185 (15.43)	
Female				**<0.01**
<80	1196 (31.57)	429 (26.40)	185 (15.43)	
≥80	1173 (30.96)	664 (40.86)	600 (50.04)	
Waist-to-hip ratio				
Male				**<0.01**
<0.90	722 (19.06)	203 (12.49)	109 (9.09)	
≥0.90	698 (18.42)	329 (20.25)	305 (25.44)	
Female				**<0.01**
<0.85	1200 (31.67)	391 (24.06)	134 (11.18)	
≥0.85	1169 (30.85)	702 (43.20)	651 (54.30)	
Education years				**<0.01**
Illiteracy	1106 (29.19)	624 (38.40)	492 (41.03)	
1–6	1384 (36.53)	559 (34.40)	418(34.86)	
7–9	930 (24.54)	334 (20.55)	210 (17.51)	
≥10	369 (9.74)	108 (6.65)	79 (6.59)	
Occupation				**<0.01**
Farmer	2642 (69.73)	1251 (76.98)	973 (81.15)	
Worker	441 (11.64)	166 (10.22)	77 (6.42)	
Service staff	271 (7.15)	76 (4.68)	44 (3.67)	
White-collar	394 (10.40)	114 (7.02)	95 (7.92)	
Other	41 (1.08)	18 (1.11)	10 (0.83)	
Smoking status				**<0.01**
Never	2735 (72.18)	1250 (76.92)	902 (75.23)	
Former	312 (8.23)	148 (9.11)	107 (8.92)	
Current	742 (19.58)	227 (13.97)	190 (15.85)	
Alcohol drinking				0.65
Never	3348 (88.36)	1451 (89.29)	1069 (89.16)	
Former	130 (3.43)	46 (2.83)	42 (3.50)	
Current	311 (8.21)	128 (7.88)	88 (7.34)	
Tea drinking				**<0.01**
Never	2850 (75.22)	1283 (78.95)	916 (76.40)	
Former	34 (0.90)	18 (1.11)	21 (1.75)	
Current	905 (23.88)	324 (19.94)	262 (21.85)	
Hypertension				**<0.01**
No	2331 (61.52)	780 (48.00)	398 (33.19)	
Yes	1458 (38.48)	845 (52.00)	801 (66.81)	
Hyperlipidemia				**<0.01**
No	2625 (69.28)	1021 (62.83)	650 (54.21)	
Yes	1164 (30.72)	604 (37.17)	549 (45.79)	
Lipid-lowering drugs				**<0.01**
No	3746 (98.87)	1596 (98.22)	1141 (95.16)	
Yes	43 (1.13)	29 (1.78)	58 (4.84)	
Total and differential WBC counts				
Total WBC count, ×109/L	5.57 (4.76, 6.55)	5.81 (4.98, 6.82)	6.21 (5.33, 7.25)	**<0.01**
Neutrophil count, ×109/L	3.04 (2.48, 3.81)	3.20 (2.59, 4.00)	3.49 (2.84, 4.31)	**<0.01**
Lymphocyte count, ×109/L	1.96 (1.62, 2.36)	2.04 (1.68, 2.47)	2.12 (1.74, 2.59)	**<0.01**
Monocyte count, ×109/L	0.33 (0.27, 0.41)	0.34 (0.28, 0.41)	0.35 (0.29, 0.43)	**<0.01**
Eosinophil count, ×109/L	0.10 (0.06, 0.16)	0.10 (0.06, 0.15)	0.10 (0.06, 0.15)	**>0.05**
Basophil count, ×109/L	0.02 (0.01, 0.03)	0.02 (0.01, 0.03)	0.02 (0.02, 0.03)	**<0.01**

Demographic and clinical characteristics among normoglycemia, prediabetes, and diabetes groups are presented in Table 1. Variates are presented as *n* (%), or median (IQR). The distribution of variables between groups was tested by the Chi-square test or Kruskal-Wallis’s test. A significant association (*P* < 0.01) is indicated in bold.

### Association between WHR and different glycemic status

As shown in Table 2, we investigated the relationship between WHR and glycemic status using logistic regression analysis. In the crude model, higher WHR was associated with an increased risk of prediabetes (*OR* = 1.79, 95% *CI*: 1.59, 2.01) and diabetes (*OR* = 4.05, 95% *CI*: 3.47, 4.73). The excessive risk slightly attenuated but still persisted after additional adjustment for potential confounders, with an *OR* of 1.53 (95% *CI*: 1.35, 1.74) for prediabetes and 2.89 (95% *CI*: 2.45, 3.40) for diabetes. In addition, the association between WHR and glycemic status were further analyzed after stratified by sex. Consistent positive association was observed in both male and female group. The *OR* was 1.61 (95% *CI*: 1.30, 1.99) and 2.58 (95% *CI*: 2.00, 3.33) for prediabetes and diabetes in male. In female, the *OR* was 1.47 (95% *CI*: 1.26, 1.72) and 3.14 (95% *CI*: 2.52, 3.90) respectively.

**Table 2:** Logistic regression models for the association of central adiposity with glycemic status.

Variables	Univariate model		Multivariate model*	
	Prediabetes	Diabetes	Prediabetes	Diabetes
	*OR* (95%*CI*)	*OR* (95%*CI*)	*OR* (95%*CI*)	*OR* (95%*CI*)
Waist-to-hip ratio				
Normal	Reference	Reference	Reference	Reference
Central adiposity	1.79 (1.59,2.01)	4.05 (3.47,4.73)	1.53 (1.35,1.74)	2.89 (2.45,3.40)
Male				
<0.90	Reference	Reference	Reference	Reference
≥0.90	1.68 (1.37,2.06)	2.89 (2.27,3.69)	1.61 (1.30,1.99)	2.58 (2.00,3.33)
Female				
<0.85	Reference	Reference	Reference	Reference
≥0.85	1.84 (1.59,2.14)	4.99 (4.07,6.11)	1.47 (1.26,1.72)	3.14 (2.52,3.90)

*Adjusted for age, education years, occupation, smoking, alcohol drinking, tea drinking, hypertension, hyperlipidemia, and lipid-lowering drugs.

Univariate logistic regression and multivariate logistic regression were applied to explore the relationship between waist-to-hip ratio and glycemic status. Results are shown for the entire study population, and separated for men and women.

### Association between WHR and levels of circulating WBCs

Spearman’s rank correlation coefficients between WHR and the levels of total WBC and the five types of WBC are listed in Table 3. Positive association was observed between WHR and levels of WBCs, correlation coefficient of which varied from 0.12 to 0.23 (all *P* < 0.01).

**Table 3:** Association between WHR and circulating white blood cells.

Variables	1	2	3	4	5	6	7
1. Waist-to-hip ratio	—						
2. Total WBC	0.23^*^	—					
3. Neutrophils	0.19^*^	0.88^*^	—				
4. Lymphocytes	0.16^*^	0.60^*^	0.20^*^	—			
5. Monocytes	0.22^*^	0.65^*^	0.52^*^	0.38^*^	—		
6. Eosinophils	0.14^*^	0.25^*^	0.11^*^	0.23^*^	0.28^*^	—	
7. Basophils	0.12^*^	0.28^*^	0.21^*^	0.21^*^	0.20^*^	0.32^*^	—

Table 3 presents the Spearman rank correlation coefficients between waist-to-hip ratio and circulating white blood cells. *P* value less than 0.01 is represented by“*”.

### Association between WBC and different glycemic status

**Fig. 1:**
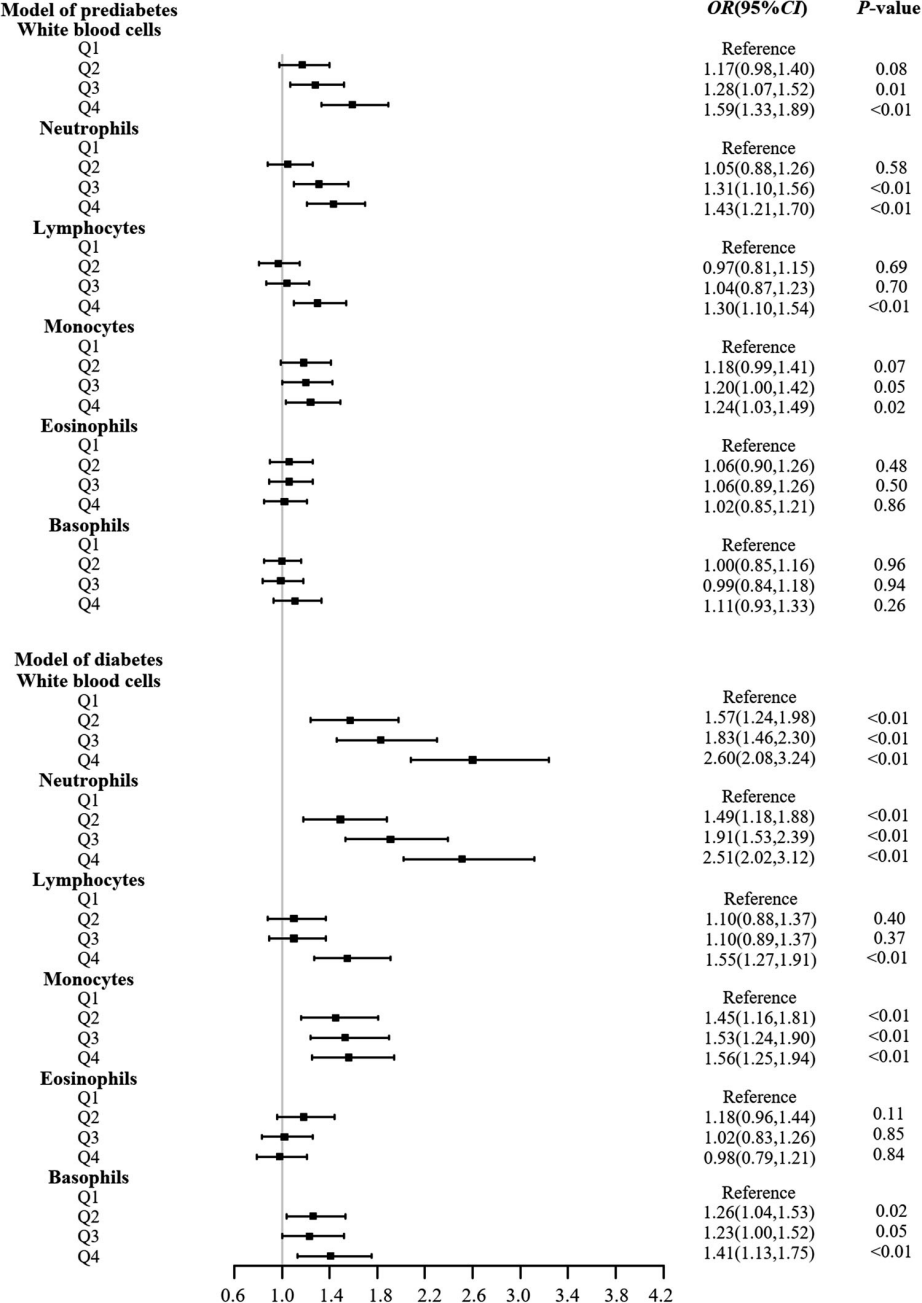
Logistic regression models for the associations between WBC counts and glycemic status. WBCs were divided into quartiles according to the distributions in the control population. Logistic regressions were performed to explore the effect of WBCs on diabetes. Analysis was adjusted for waist-to-hip ratio, age, sex, education, occupation, smoking, alcohol drinking, tea drinking, hypertension, hyperlipidemia, and lipid-lowering drugs.

In addition, the association between WBC and glycemic status was analyzed after adjusting for WHR and other covariates mentioned earlier and the results were shown in Fig. 1. Elevated peripheral WBC counts, including total white blood cell, neutrophil, lymphocyte, and monocyte, were associated with increased risks of prediabetes or diabetes. However, no significant correlation was observed between eosinophil or basophil and glycemic status.

### Mediation analyses

In the mediation analysis, we explore how much of the association between WHR and glycemic status was mediated through WBC counts. We examined the mediation effect of each type of WBC separately in the simple mediation model, and the overall mediation effect through all five types of WBC simultaneously in the multiple mediation model.

### Simple mediation model for prediabetes and diabetes

Simple mediation analysis results are depicted in the top rows of Table 4. After covariate adjustment, statistically significant indirect effects were observed for total WBC, neutrophil, lymphocyte, and monocyte counts when they were individually included as mediators, indicating that these variables by themselves mediated the association between WHR and prediabetes. The proportions mediated by these four indicators were 9.92%, 6.98%, 6.07%, and 3.84%, respectively. Similarly, for the association between central adiposity and diabetes risk, significant mediation was also observed by total WBC count, neutrophil count, lymphocyte count, monocyte count, and basophil count, with a mediated proportion of 11.79%, 11.51%, 6.29%, 4.78%, and 1.76%, respectively.

**Table 4:** Mediation analysis of WBC counts for the association between central adiposity and glycemic status.

Mediator(s)	Natural direct effect		Natural indirect effect		Total effect		Proportion mediated
	*OR* (95% *CI*)	*P* value	*OR* (95% *CI*)	*P* value	*OR* (95% *CI*)	*P* value	
Model of prediabetes^a^							
Total WBC	1.46 (1.29,1.65)	<0.01	1.03 (1.02,1.06)	<0.01	1.51 (1.34,1.72)	<0.01	9.92
Neutrophils	1.48 (1.31,1.68)	<0.01	1.02 (1.01,1.05)	<0.01	1.51 (1.34,1.72)	<0.01	6.98
Lymphocytes	1.48 (1.32,1.69)	<0.01	1.02 (1.01,1.04)	<0.01	1.51 (1.34,1.72)	<0.01	6.07
Monocytes	1.49 (1.32,1.70)	<0.01	1.01 (1.00,1.03)	0.04	1.51 (1.34,1.72)	<0.01	3.84
Eosinophils	1.51 (1.34,1.72)	<0.01	1.00 (0.99,1.01)	0.64	1.52 (1.34,1.72)	<0.01	NA
Basophils	1.51 (1.33,1.72)	<0.01	1.00 (1.00,1.01)	0.63	1.51 (1.34,1.72)	<0.01	NA
All five types of WBC^c^	1.46 (1.29,1.65)	<0.01	1.04 (1.02,1.07)	<0.01	1.51 (1.34,1.72)	<0.01	10.09
Model of diabetes^b^							
Total WBC	2.46 (2.11,2.89)	<0.01	1.08 (1.06,1.13)	<0.01	2.65 (2.31,3.14)	<0.01	11.79
Neutrophils	2.50 (2.14,2.94)	<0.01	1.08 (1.05,1.11)	<0.01	2.69 (2.30,3.15)	<0.01	11.51
Lymphocytes	2.58 (2.22,3.04)	<0.01	1.04 (1.02,1.07)	<0.01	2.69 (2.30,3.15)	<0.01	6.29
Monocytes	2.60 (2.23,3.06)	<0.01	1.03 (1.01,1.06)	<0.01	2.68 (2.30,3.16)	<0.01	4.78
Eosinophils	2.68 (2.30,3.16)	<0.01	1.00 (0.98,1.01)	0.65	2.67 (2.30,3.15)	<0.01	NA
Basophils	2.63 (2.26,3.09)	<0.01	1.01 (1.00,1.03)	0.01	2.66 (2.29,3.13)	<0.01	1.76
All five types of WBC^c^	2.46 (2.12,2.90)	<0.01	1.09 (1.06,1.14)	<0.01	2.68 (2.31,3.18)	<0.01	12.77

^a^Predictor (central adiposity vs. normal); mediator (white blood cells, neutrophils, lymphocytes, monocytes, eosinophils, or basophils); outcome (prediabetes vs. normorglycemia).

^b^Predictor (central adiposity vs. normal); mediator (white blood cells, neutrophils, lymphocytes, monocytes, eosinophils, or basophils); outcome (diabetes vs. normorglycemia).

^c^Five types of WBC including neutrophils, lymphocytes, monocytes, eosinophils, and basophils were included in the model simultaneously.

Each analytical step was adjusted for age, sex, education years, occupation, smoking, alcohol drinking, tea drinking, hypertension, hyperlipidemia, and lipid-lowering drugs.

Both simple and parallel multiple mediation models were used to explore the potential mediation effects of WBCs on the association of waist-to-hip ratio with diabetes. Each analytical step was adjusted for age, sex, education years, occupation, smoking, alcohol drinking, tea drinking, hypertension, hyperlipidemia, and lipid-lowering drugs.

### Multiple mediation model for prediabetes and diabetes

To further determine the overall contribution of different types of WBC to the relationship between central adiposity and glycemic status, all five types of WBC were included simultaneously and multiple mediation analysis was conducted. The results for models pertaining to prediabetes and diabetes were presented in the last row of Table 4, separately. In the multiple mediator model for prediabetes, a statistically significant indirect effect was observed confirming the overall mediating role of the five types of WBC combined in the relation between central adiposity and risk of prediabetes (*OR* = 1.04, 95% CI: 1.02, 1.07), with a total mediated proportion of 10.09%.

In the multiple mediator model for diabetes, we found significant indirect effect (*OR* = 1.09, 95%*CI* = 1.06 to 1.14) for combined WBCs. 12.77% of the total effect of central adiposity on diabetes was jointly mediated by five types of white blood cells.

### Sensitivity analysis

We also performed a sensitivity analysis after excluding the participants who reported use of antibiotic drugs and whose total white blood cell count was below the 2.5th percentile and above 97.5th percentile (*n* = 4374). Result and conclusion sustained in the sensitivity analysis (Supplementary Table 1–3, Supplementary Fig. 2). Elevated peripheral WBC counts, including total white blood cell, neutrophil, lymphocyte and monocyte, were associated with increased risks of prediabetes or diabetes (Supplementary Fig. 2). The total white blood cells count was a significant mediator of the association of central adiposity with diabetes, which mediating 15.03% of the total effect. The portion of the total effect that was mediated through five different types of white blood cells was found to be 11.71% and statistically significant (Supplementary Table 3).

## Discussion

The purpose of the present study was to examine whether the increase in waist-to-hip ratio exerts effects on white blood cells and whether white blood cells mediated the effects of central adiposity on diabetes. Results indicated that waist-to-hip ratio was positively linked to the total WBC and differential counts of WBC, which was in line with published studies demonstrating relationship between obesity and systemic inflammation [20, 21]. In addition to the observed relation between WBC counts and diabetes, results of the simple and parallel multiple mediation models extended previous findings by elucidating that the impact of waist-to-hip ratio on diabetes is mediated by white blood cells.

Large scale prospective studies of varied populations have revealed the association between central adiposity and glycemic status. Wang et al. compared the causal effect of overall obesity and abdominal obesity on type 2 diabetes among Chinese Han individuals [22]. They found that higher WHR increased the risk of glucose deterioration, whereas no significant association of BMI with glucose deterioration was observed, which suggested that WHR may be more sensitive than BMI in the prediction of diabetes. In another large prospective cohort of 0.5 million middle-to-older-aged Chinese people, it was reported that maintaining a lower WHR was associated with a significantly reduced risk of diabetes [23]. Similar findings are also reported in a recent meta-analysis, in which each increase in waist-to-hip ratio by 0.1 units was linked to a 63% higher risk of type 2 diabetes (relative risk 1.63, 95% confidence interval 1.50 to 1.78, I^2^ = 99%) [24].

Low-grade inflammation is a key component in the pathophysiology of type 2 diabetes, particularly in the development of obesity-related insulin resistance. A growing body of evidence support that obesity leads to overexpression of proinflammatory adipokines, which can activate various signal transduction cascades, including pathways that are regarded as critical inhibitors of insulin action and pancreatic function [25, 26]. Leukocyte infiltration is an important feature of inflammatory response in obesity [27, 28]. Animal studies have shown that obesity-induced inflammatory responses involve systemic increases in circulating inflammatory cytokines including IL-6 and IL-8, which are potent WBC proliferation and differentiation factors [29–32]. White blood cell count, a marker of subclinical inflammation, is directly associated with insulin resistance and inversely with insulin secretion. White blood cell count has been reported to predict both worsening insulin sensitivity and incident of type 2 diabetes. A multi-center observational epidemiologic study conducted by Lorenzo et al. reported that elevated total white blood cell, neutrophil, and lymphocyte counts were detected in individuals who are at increased risk of diabetes [33]. Wan et al. reported that increased levels of total WBC count, neutrophil count, lymphocyte count, monocyte count, eosinophil count, and basophil count were related to an elevated risk of diabetes [34].

Thus it is plausible to speculate that elevated WBC plays a crucial role in the development of diabetes attributed to obesity. A similar hypothesis was previously suggested by Lewis et al. [35]. They found that people with obesity and increased WBC count were at elevated risk of developing diabetes using multivariate Cox proportional hazards regression models and further revealed that WBC count was a stratifying factor in the prediction of diabetes risk among individuals with obesity. However, how and to what extent WBC counts affect the association of obesity and diabetes remains unclear. Another cohort study from the Korean population explored the predictive value of white blood cell count in incident Type 2 diabetes mellitus among adults without obesity, and found that the higher quartile of WBC count groups showed significantly higher cumulative T2DM incidence over 10 years [36]. However, the potential role of white blood cells in obese adults and if the role of white blood cells differed between adults with and without obesity has not been further elucidated.

The present study used mediation analysis to provide more robust evidence of the mediating role of WBC in the association between obesity and diabetes. In the simple mediation analysis, we found that associations between central obesity can be mediated by total WBC count by the proportion of 9.92% for prediabetes and 11.79% for diabetes, respectively. In addition, we also observed significant independent mediation effects for neutrophil, lymphocyte, and monocyte. However, no independent mediating effect of eosinophils and basophils was found. Considering the possibility of interactions among component effects of different types of WBCs [37], we therefore conducted parallel multiple mediation models to examine the role of all five types of WBCs simultaneously on diabetes. We find that the total indirect effect of all five types of WBC were 12.77% in the parallel multiple mediation models of diabetes, which was close to that of directly using the total WBC count in the simple mediation model for diabetes, which indicated independent mediating effect of the five types of white blood cells in regulating inflammation in diabetes.

The study has several limitations. First, the cross-sectional design does not allow the assessment of temporal relationships between central adiposity, WBC, and glycemic status. Second, information on drug use and comorbidity was absent in the presented study. The use of steroids and nonsteroidal anti-inflammatory drugs, previous use of antibiotics and therapy durations, and information about other diseases that could cause chronic inflammation, such as periodontal disease, all of which may possibly influence leukocyte counts. However, our analysis limited the inclusion of participants with extremely abnormal levels of inflammation, adjusted for a majority of covariates that may affect blood cell counts, particularly smoking status, drinking status, and accompanying diseases (e.g. hypertension and hyperlipidemia). Third, we cannot exclude the possibility that other variables could also mediate the associations between central obesity and diabetes.

In conclusion, central obesity was positively associated with the risk of diabetes. Mediation analysis suggested that the level of circulating total and differential WBC may be one of the key underlying mechanisms through which central obesity is associated with worsening glycemic status.

### Supplementary information

Supplementary table 1

Supplementary table 2

Supplementary table 3

Supplementary figure 1

Supplementary figure 2

Supplementary figure legend

## Data Availability

The anonymized datasets generated during and/or analyzed during the current study are available from the corresponding author upon reasonable request.
