# Supplementary material for: The role of peripheral white blood cell counts in the association between central adiposity and glycemic status
Source: Nutr Diabetes. 2024 May 17;14:30. doi: 10.1038/s41387-024-00271-9 (PMC11101409; doi:10.1038/s41387-024-00271-9)
Supplement: Supplementary file 1 — Supplementary table 1 [file 41387_2024_271_MOESM1_ESM.docx]

Supplementary table 1 Logistic regression models for the association of central adiposity with glycemic status in sensitivity analysis

| Variables | Univariate model | |  | Multivariate model* | |
| --- | --- | --- | --- | --- | --- |
|  | Prediabetes | Diabetes |  | Prediabetes | Diabetes |
|  | *OR*(95%*CI*) | *OR*(95%*CI*) |  | *OR*(95%*CI*) | *OR*(95%*CI*) |
| Waist-to-hip ratio |  |  |  |  |  |
| Normal | Reference | Reference |  | Reference | Reference |
| Central adiposity | **1.85(1.60,2.15)** | **3.96(3.27,4.79)** |  | **1.62(1.39,1.89)** | **2.89(2.36,3.53)** |
| Male |  |  |  |  |  |
| <0.90 | Reference | Reference |  | Reference | Reference |
| ≥0.90 | **1.73(1.35,2.21)** | **3.21(2.38,4.34)** |  | **1.73(1.34,2.23)** | **2.98(2.17,4.09)** |
| Female |  |  |  |  |  |
| <0.85 | Reference | Reference |  | Reference | Reference |
| ≥0.85 | **1.92(1.60,2.31)** | **4.50(3.52,5.75)** |  | **1.55 (1.27,1.90)** | **2.91(2.23,3.80)** |
| * Adjusted for age, education years, occupation, smoking, alcohol drinking, tea drinking, hypertension, hyperlipidemia and lipid-lowering drugs. | | | | | |

Table legend

Supplementary table 1 Logistic regression models for the association of central adiposity with glycemic status in sensitivity analysis

After excluding the participants who reported use of antibiotic drugs and whose total white blood cells count was below the 2.5th percentile and above 97.5th percentile, 4374 subjects remained. Univariate logistic regression and multivariate logistic regression were applied to explore the relationship between waist-to-hip ratio and glycemic status in these populations.
