# Supplementary material for: The role of peripheral white blood cell counts in the association between central adiposity and glycemic status
Source: Nutr Diabetes. 2024 May 17;14:30. doi: 10.1038/s41387-024-00271-9 (PMC11101409; doi:10.1038/s41387-024-00271-9)
Supplement: Supplementary file 2 — Supplementary table 2 [file 41387_2024_271_MOESM2_ESM.docx]

Supplementary table 2 Association between WHR and circulating white blood cells in sensitivity analysis

| Variables | 1 | 2 | 3 | 4 | 5 | 6 | 7 |
| --- | --- | --- | --- | --- | --- | --- | --- |
| 1.Waist-to-hip ratio | - |  |  |  |  |  |  |
| 2.Total WBC | 0.21^*^ | - |  |  |  |  |  |
| 3.Neutrophils | 0.16^*^ | 0.86^*^ | - |  |  |  |  |
| 4.Lymphocytes | 0.15^*^ | 0.57^*^ | 0.13^*^ | - |  |  |  |
| 5.Monocytes | 0.21^*^ | 0.59^*^ | 0.45^*^ | 0.33^*^ | - |  |  |
| 6.Eosinophils | 0.12^*^ | 0.25^*^ | 0.10^*^ | 0.21^*^ | 0.28^*^ | - |  |
| 7.Basophils | 0.12^*^ | 0.27^*^ | 0.19^*^ | 0.18^*^ | 0.17^*^ | 0.32^*^ | - |

Supplementary table 2 Association between WHR and circulating white blood cells in sensitivity analysis

Associations between waist-to-hip ratio and circulating white blood cells were evaluated using Spearman’s rank correlation analysis. *P* value less than 0.01 is represented by“*”.
