# Supplementary material for: The role of peripheral white blood cell counts in the association between central adiposity and glycemic status
Source: Nutr Diabetes. 2024 May 17;14:30. doi: 10.1038/s41387-024-00271-9 (PMC11101409; doi:10.1038/s41387-024-00271-9)
Supplement: Supplementary file 3 — Supplementary table 3 [file 41387_2024_271_MOESM3_ESM.docx]

Supplementary table 3 Mediation analysis of WBC counts for the association between central adiposity and glycemic status in sensitivity analysis

| Mediator(s) | Natural direct effect | |  | Natural indirect effect | |  | Total effect | | Proportion  mediated |
| --- | --- | --- | --- | --- | --- | --- | --- | --- | --- |
|  | *OR*(95% *CI*) | *P* value |  | *OR*(95% *CI*) | *P* value |  | *OR*(95% *CI*) | *P* value |  |
| Model of prediabetes† |  |  |  |  |  |  |  |  |  |
| Total WBC | 1.54(1.32,1.80) | <0.01 |  | 1.03(1.02,1.07) | <0.01 |  | 1.59(1.37,1.87) | <0.01 | 8.47 |
| Neutrophils | 1.57(1.35,1.84) | <0.01 |  | 1.02(1.01,1.04) | 0.01 |  | 1.60(1.38,1.87) | <0.01 | 5.47 |
| Lymphocytes | 1.57(1.36,1.84) | <0.01 |  | 1.02(1.00,1.04) | 0.03 |  | 1.60(1.38,1.87) | <0.01 | 5.00 |
| Monocytes | 1.57(1.34,1.83) | <0.01 |  | 1.02(1.01,1.05) | 0.01 |  | 1.60(1.38,1.87) | <0.01 | 6.33 |
| Eosinophils | 1.60(1.37,1.86) | <0.01 |  | 1.00(0.99,1.01) | 0.79 |  | 1.60(1.37,1.86) | <0.01 | NA |
| Basophils | 1.60(1.37,1.87) | <0.01 |  | 1.00(0.99,1.01) | 0.96 |  | 1.60(1.37,1.87) | <0.01 | NA |
| All five types of WBC ^a^ | 1.55(1.33,1.81) | <0.01 |  | 1.03(1.01,1.07) | 0.02 |  | 1.60(1.38,1.87) | <0.01 | 8.12 |
| Model of diabetes‡ |  |  |  |  |  |  |  |  |  |
| Total WBC | 2.48(2.06,3.05) | <0.01 |  | 1.11(1.06,1.14) | <0.01 |  | 2.74(2.25,3.33) | <0.01 | 15.03 |
| Neutrophils | 2.55(2.11,3.11) | <0.01 |  | 1.07(1.03,1.10) | <0.01 |  | 2.73(2.24,3.31) | <0.01 | 10.08 |
| Lymphocytes | 2.64(2.18,3.23) | <0.01 |  | 1.02(1.01,1.06) | 0.01 |  | 2.70(2.24,3.32) | <0.01 | 3.56 |
| Monocytes | 2.63(2.17,3.22) | <0.01 |  | 1.03(1.01,1.06) | 0.01 |  | 2.71(2.24,3.32) | <0.01 | 4.59 |
| Eosinophils | 2.72(2.24,3.32) | <0.01 |  | 1.00(0.98,1.01) | 0.57 |  | 2.71(2.23,3.30) | <0.01 | NA |
| Basophils | 2.67(2.22,3.26) | <0.01 |  | 1.01(1.00,1.03) | 0.07 |  | 2.70(2.24,3.30) | <0.01 | NA |
| All five types of WBC ^a^ | 2.52(2.07,3.08) | <0.01 |  | 1.08(1.04,1.14) | <0.01 |  | 2.72(2.25,3.33) | <0.01 | 11.71 |

†Predictor (central adiposity vs. normal); mediator ( white blood cells, neutrophils, lymphocytes, monocytes, eosinophils or basophils); outcome ( prediabetes vs. normorglycemia)

‡Predictor (central adiposity vs. normal); mediator ( white blood cells, neutrophils, lymphocytes, monocytes, eosinophils or basophils); outcome ( diabetes vs. normorglycemia)

^a^ Five types of WBC including neutrophils, lymphocytes, monocytes, eosinophils and basophils were included in the model simultaneously.

Each analytical step was adjusted for age, sex, education years, occupation, smoking, alcohol drinking, tea drinking, hypertension, hyperlipidemia and lipid-lowering drugs.

Table legend

Supplementary table 3 Mediation analysis of WBC counts for the association between central adiposity and glycemic status in sensitivity analysis

Both simple and parallel multiple mediation models were used to explore the potential mediation effects of WBCs on the association of waist-to-hip ratio with diabetes. Each analytical step was adjusted for age, sex, education years, occupation, smoking, alcohol drinking, tea drinking, hypertension, hyperlipidemia and lipid-lowering drugs.
