# Supplementary figures and images for: The role of peripheral white blood cell counts in the association between central adiposity and glycemic status
Source: Nutr Diabetes. 2024 May 17;14:30. doi: 10.1038/s41387-024-00271-9 (PMC11101409; doi:10.1038/s41387-024-00271-9)

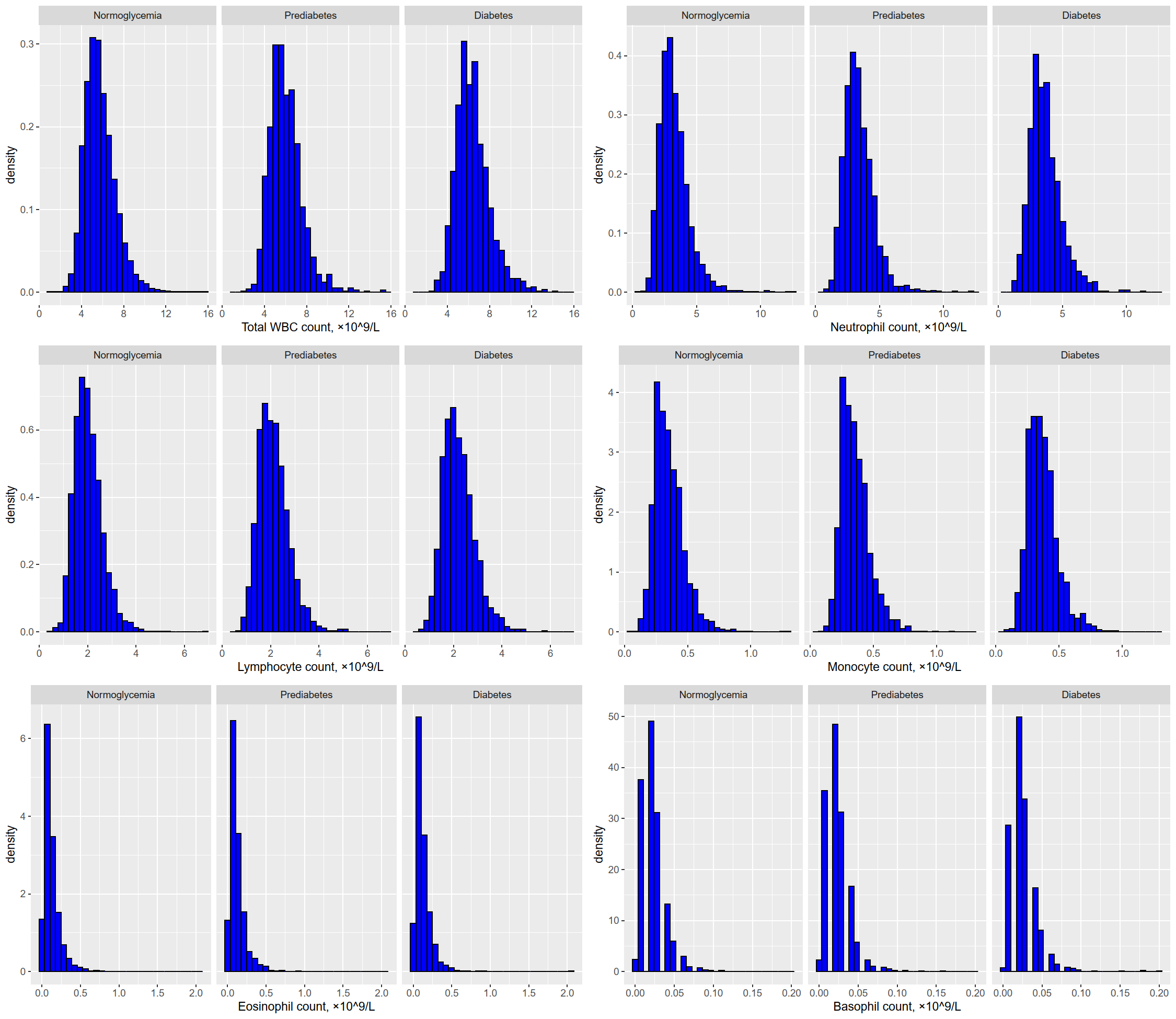

Supplement: Supplementary file 4 — Supplementary figure 1 [file 41387_2024_271_MOESM4_ESM.png]

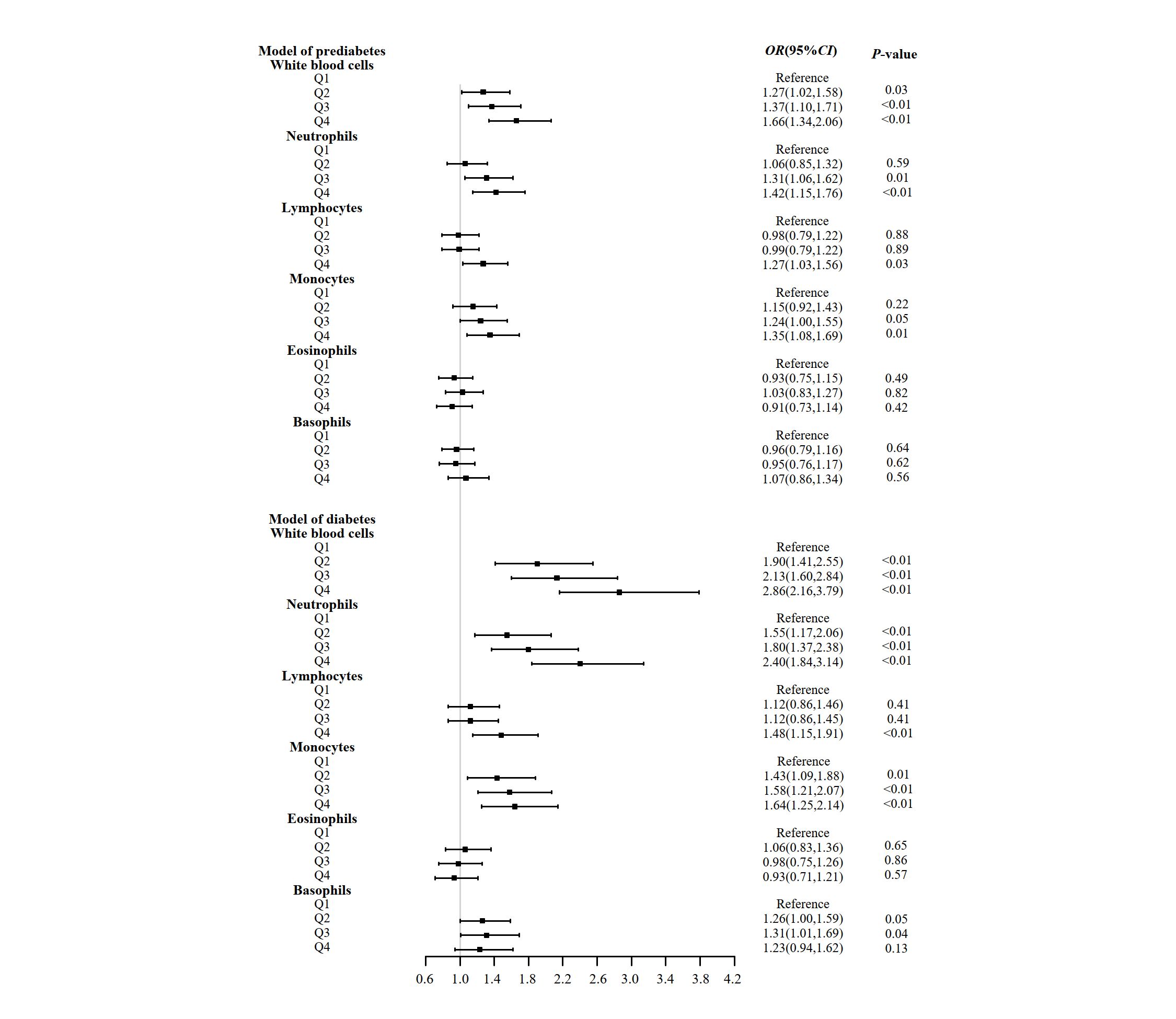

Supplement: Supplementary file 5 — Supplementary figure 2 [file 41387_2024_271_MOESM5_ESM.png]
