# Supplementary material for: The role of peripheral white blood cell counts in the association between central adiposity and glycemic status
Source: Nutr Diabetes. 2024 May 17;14:30. doi: 10.1038/s41387-024-00271-9 (PMC11101409; doi:10.1038/s41387-024-00271-9)
Supplement: Supplementary file 6 — Supplementary figure legend [file 41387_2024_271_MOESM6_ESM.docx]

**Supplementary figure legend**

**Supplementary Figure 1 Distribution of white blood cell among normoglycemia, prediabetes, and diabetes groups**

Supplementary Figure 1 depicts the distribution of total and differential white blood cell counts in the total study population

**Supplementary Figure 2 Logistic regression models for the associations between WBC counts and glycemic status.**

WBCs were divided into quartiles according to the distributions in the control population. Logistic regressions were performed to explore the effect of WBCs on diabetes. Analysis was adjusted for waist-to-hip ratio, age, sex, education, occupation, smoking, alcohol drinking, tea drinking, hypertension, hyperlipidemia and lipid-lowering drugs.
